# Species-specific bioaccumulation of trace metals among fish species from Xincun Lagoon, South China Sea

**DOI:** 10.1038/s41598-020-77917-y

**Published:** 2020-12-11

**Authors:** Weihua Feng, Zhifu Wang, Hengtao Xu, Dongrong Zhang, Haifeng Zhang, Wenzhuo Zhu

**Affiliations:** 1 Key Laboratory of Engineering Oceanography, Second Institute of Oceanography, Ministry of Natural Resource of the People’s Republic of China, Hangzhou, 310012 China; 2 College of Marine Science and Technology, Zhejiang Ocean University, Zhoushan, 316022 China

**Keywords:** Biochemistry, Environmental sciences, Ocean sciences

## Abstract

Xincun Lagoon is an important fishing area in northern Hainan Island, China. It has long been exposed to pollutants from local sewage, breeding cages and fishing boats, resulting in serious pollution threats to the survival of fish. In this study, we examined the concentrations of seven trace metals (Cu, Pb, Zn, Cd, Hg, As and Cr) in sixteen economic fish species collected from Xincun Lagoon and their exposed environment (water and sediment). The concentrations of Pb and Zn in the water column were higher than the safety threshold stipulated by Chinese legislation, while the contents of all examined metals in the sediment and fish species were lower than the legislative thresholds set by China and international organizations. The contents of trace metals in the fish species in high trophic levels or those that prefer to live in/on the sediment layer were significantly higher than those in other trophic levels and pelagic/subbenthic fish, these species had homogeneous anthropogenic pollution sources for Cu, Zn, Cd, Hg and As. Our results show that the trace metal contents in fish were mainly affected by trophic level and habitat preference. The contribution of metal exposure from the sediment to metal accumulation in fish was lower than that in water, especially for the Cd and Hg in the sediment. These results provide valuable information for further understanding the species-specific patterns of metal accumulation in fish and the development of targeted conservation measures for the environment and fish consumers.

## Introduction

Trace metals are released into the ocean from both natural sources (e.g. volcanism, rock erosion, and atmospheric deposition) and anthropogenic sources (e.g. industrial activities, river transportation, shipping, and aquaculture)^1,2^. In coastal sea areas, metals are mainly contributed by human activities^3^. In recent decades, the problem of metal pollution in coastal seas has attracted increasing attention, not only because it can reduce the structural function of marine ecosystems, but also because of the health hazards to humans who like to consume seafood^4^. Trace metals seriously threaten the survival of marine life and the food safety of coastal people due to their toxicity, persistence, bioaccumulation and bioamplification in the food chain^4–7^. Biologically, copper (Cu) and zinc (Zn) are important essential elements and play an important role in the metabolism of living organisms^8^. However, they can also produce toxicity in organisms when their contents exceed the safety thresholds, so changes in the contents of these metals in organisms and in their environment require monitoring^9,10^. Cadmium (Cd), lead (Pb), arsenic (As) and mercury (Hg) are nonessential biological metals that exhibit extreme toxicity, even at trace levels^11^. For instance, cadmium accumulation in the human body can induce skeletal damage, kidney dysfunction and reproductive deficiencies^12,13^; Lead can significantly reduce semen quality, thus impairing human reproductive function^14^; Arsenic can cause toxic and harmful biological effects such as liver, skin and bladder cancer^15^; Mercury can cause adverse effects such as impair hearing, eyesight, and the nervous system^8,16^. The biological function of chromium is unclear, but when its concentration exceeds the threshold, it can cause damage to the liver, kidney, lungs and is recognized as a known carcinogen^9,17^.

Among marine organisms, fish are the most common indicator of environmental pollution as they occupy a range of trophic levels in the food chain^8,18,19^. Fish are an important source of nutrition for human beings because they are rich in protein, lipids, vitamins and minerals^20–23^. Although humans can obtain nutrients from fish consumption, contaminated fish pose a potential health risk to higher trophic level organisms, such as humans, that may consume them, so it is important to evaluate the levels of metal contaminants in fish^24,25^.

Studies have found the accumulation of metals in fish tissues are mainly dependent upon the exposure period and metal concentrations the fish are subjected too. Additionally, the physiological needs of the fish, its sex and size have also been shown to effect metal accumulation in their tissues^13,26–28^. However, metal transfer among biogeochemical compartments, accumulation in organisms, and bioamplification in food webs depend on the food composition and concentrations of metals in food and habitats^2,29^. Therefore, understanding the mechanisms that lead to the bioaccumulation of trace metals in consumers and explaining their metal burden requires a good knowledge of the relationship between feeding habits, trophic ecology and metal accumulation.

Xincun Lagoon is an important fishing area in the south of Hainan Island. Currently, a large number of breeding cages account for approximately a quarter of this lagoon. A dozen cages are used in series to form fishing rows, with hundreds of fishing rows in the same breeding area, resulting in a decrease in water movement and reduced exchange capacity. In the past 30 years, the pollutants produced in the process of cage farming have been released into the water continuously, resulting in increasingly serious environmental pollution in the water of the Xincun Lagoon^30^. At the same time, this lagoon is also an important national fishing port, where a large number of fishing boats dock all year round. Although the environment of Xincun Lagoon is generally deteriorating, there are few reports on metal monitoring and attention to possible increases in metals concentrations in this area.

The specific objectives of our study are as follows: (1) to describe the contents of seven trace metals (Cu, Pb, Zn, Cd, Hg, As and Cr) in sixteen common economic fish species and their habitats (water and sediment) in Xincun Lagoon; (2) to assess the burden and food safety of trace metals in fish; (3) to explore the potential relationship between trophic levels, habitat preferences of fish and trace metal accumulation; and (4) to reveal the specific-specific bioaccumulation patterns of the trace metals.

## Results and discussion

### Trace metal concentrations in water and sediment

Trace metal concentrations in water and sediment samples from Xincun Lagoon provided the background value for the fish living environment. The concentrations of the study metals in water and sediment are presented in Supplementary Tables 1 and 2, respectively, together with the legislative thresholds. The highest zinc concentration (62.7 µg·L^−1^) in the water was 3 times higher than that specified in the Seawater Quality Standard of China (SQSC, 20 µg·L^−1^). In fact, the Zn levels from most sites were higher than those listed in the SQSC, indicating serious Zn contamination in the water columns of Xincun Lagoon. The high concentrations of zinc in Xincun Lagoon may be attributed to exogenous inputs, such as coastal domestic sewage, domestic waste, shipping activities and cage culture of marine fish (a method of producing fish by placing cages made of mesh pieces in sea water). In addition, according to the field investigation, there are more than 700 families from a culture known as the “Danjia people” living on the water surface of Xincun Lagoon. Their activities cause some water pollution, with possible high concentrations of zinc from ship anticorrosion paint^31,32^. The lead concentrations at sites X1–X3 and X15 were also higher than the legislative threshold (1 µg·L^−1^). Sites X01–X03 may be affected by the runoff of a small river located at the top of Xincun Lagoon. There are many aquatic products processing plants along the river, resulting in a high lead content in the water body of the estuary. The high concentration of lead at X15 site may be related to the nearby gas station on the water surface, due to the discharge of certain diesel oil containing lead into the sea water during refueling or driving^31,32^.

These contents (µg·g^−1^ d.w.) of Cu, Pb, Zn, Hg, As and Cr in the sediment were lower than the primary standard levels specified in the Marine Sediment Quality Standard of China (MSQSC) and the probable effect level (PEL) in the Canadian Sediment Quality Guidelines (CSQG). But the Cd contents at sites X06, X09, X11 and X12 were above the primary standard level in the MSQSC. This may be due to the fact that site X11 is located at the mouth of a small river which receives a large amount of sewage and garbage from Xincun Town, and that the sediments at sites X06, X09 and X12 contain a large amount of aquaculture feed which mainly comes from the aquaculture breeding cages area of X13–X18 sites under the action of water flowing into the lagoon, and that the sediment of these sites have relatively strong metal adsorption capacity, because of their smaller particle size, compared with other sites in the study area^33,34^.

### Variability of trace metal contents in fish species

The trace metal contents in the sixteen fish species from Xincun Lagoon are shown in Table 1. The contents of the studied trace metals in fish were much lower than the legislative thresholds established by the European Union and China^35,36^. This result indicated that trace metal contents in all studied fish species from Xincun Lagoon were relatively low and would be safe for consumption. It was obvious that the contents of Zn and Cu were higher than those of other metals, which may be due to these essential elements being required by biological organisms for various metabolic and physiological processes in life activities^8^. In addition, each metal had a wide range of contents among different fish species. For example, the Cu content in *Acipenser sinensis* was 100 times higher than that in *Trachurus japonicus*. This may be attributable to fish species which are from different trophic levels and different habitat preferences and subsequently metabolic processes which absorb and utilize these metals differently.

**Table 1:** Trace metal contents (µg·g^−1^, w.w.) in sixteen fish species (mean ± SD) collected from Xincun Lagoon, South China Sea and the legislation thresholds (LT).

Trophic guild	Habitat	Species	Cu	Pb	Zn	Cd	Hg	As	Cr
Low carnivore	Pelagic	*L. argentimaculatus*	0.19 ± 0.05	0.038 ± 0.001	4.77 ± 1.08	0.025 ± 0.003	0.0073 ± 0.0013	0.15 ± 0.01	0.035 ± 0.004
		*P. maruadsi*	0.41 ± 0.08	0.034 ± 0.006	3.98 ± 0.57	0.014 ± 0.005	0.0056 ± 0.0008	0.10 ± 0.04	0.028 ± 0.005
		*S. chinensis*	0.06 ± 0.02	0.034 ± 0.003	3.35 ± 0.22	0.013 ± 0.005	0.0019 ± 0.0002	0.13 ± 0.01	0.030 ± 0.013
		*N. gronovii*	0.04 ± 0.01	0.042 ± 0.007	3.78 ± 0.55	0.040 ± 0.005	0.0032 ± 0.0008	0.29 ± 0.04	0.096 ± 0.011
	Sub-benthic	*A. argentatus*	0.05 ± 0.02	0.033 ± 0.004	3.35 ± 0.55	0.037 ± 0.005	0.0027 ± 0.0004	0.20 ± 0.06	0.076 ± 0.009
		*T. japonicus*	0.03 ± 0.02	0.031 ± 0.004	3.83 ± 0.26	0.013 ± 0.003	0.0021 ± 0.0006	0.25 ± 0.04	0.027 ± 0.008
		*P. anomala*	0.14 ± 0.03	0.039 ± 0.006	3.63 ± 0.63	0.037 ± 0.005	0.0038 ± 0.0008	0.29 ± 0.07	0.067 ± 0.016
	Demersal	*L. brevirostris*	0.40 ± 0.08	0.046 ± 0.008	5.59 ± 0.68	0.050 ± 0.011	0.0080 ± 0.0010	0.51 ± 0.01	0.081 ± 0.015
		*U. bensasi*	0.29 ± 0.07	0.039 ± 0.004	5.15 ± 0.57	0.026 ± 0.007	0.0085 ± 0.0010	0.52 ± 0.06	0.074 ± 0.010
		*B. novae-zeelandiae*	0.63 ± 0.11	0.075 ± 0.012	6.04 ± 0.86	0.078 ± 0.013	0.0064 ± 0.0007	0.27 ± 0.04	0.109 ± 0.012
Middle carnivore	Pelagic	*C. dorab*	0.20 ± 0.01	0.025 ± 0.002	4.70 ± 0.06	0.085 ± 0.016	0.0059 ± 0.0004	0.30 ± 0.02	0.102 ± 0.013
	Sub-benthic	*G. filamentosus*	0.41 ± 0.11	0.091 ± 0.018	6.48 ± 0.76	0.034 ± 0.009	0.0051 ± 0.0007	0.13 ± 0.04	0.084 ± 0.011
	Demersal	*C. sinicus*	0.72 ± 0.10	0.142 ± 0.012	8.19 ± 0.78	0.080 ± 0.009	0.0082 ± 0.0009	0.44 ± 0.07	0.118 ± 0.016
High carnivore	Sub-benthic	*T. lepturus*	1.25 ± 0.13	0.086 ± 0.021	7.59 ± 1.12	0.146 ± 0.035	0.0068 ± 0.0011	0.65 ± 0.06	0.143 ± 0.023
		*T. hypargyreus*	1.61 ± 0.18	0.078 ± 0.011	7.94 ± 0.81	0.154 ± 0.007	0.0092 ± 0.0008	0.79 ± 0.07	0.142 ± 0.020
	Demersal	*A. sinensis*	3.65 ± 0.62	0.065 ± 0.012	19.4 ± 3.01	0.152 ± 0.037	0.0135 ± 0.0019	0.90 ± 0.22	0.273 ± 0.039
Average			0.63 ± 0.92	0.056 ± 0.031	6.11 ± 3.90	0.062 ± 0.050	0.0061 ± 0.0031	0.37 ± 0.02	0.093 ± 0.061
LT			40^a^	0.5^a^	100^a^	0.5^b^	0.5^b^	6^b^	2^a^

^a^China National Standards (2017).

^b^European Commission (2006).

We found that the highest contents of Cu, Zn, Hg, As and Cr were examined in *A. sinensis*, the highest content of Pb in *Cynoglossus sinicus* and the highest content of Cd in *Thamnaconus hypargyreus* (Table 1). In contrast, the lowest contents of Cu and Cr were detected in *T. japonicas*; the lowest content of Pb in *Chirocentrus dorab*; the lowest contents of Zn, Cd and Hg in *Stolephorus chinensis*; the lowest content of As in *Pecapterus maruadsi*. It was obvious that the highest contents of most of trace metals tended to be found in fish species that are high-trophic-guild carnivores and that prefer to live on the bottom (e.g. *A. sinensis*). In contrast, the lowest contents of most of trace metals tended to appear in the low-trophic-guild carnivorous fish, pelagic fish (e.g. *P. maruadsi* and *S. chinensis*) and sub-benthic fish (e.g. *A. argentatus* and *T. japonicus*). In general, low-trophic-guild carnivorous, pelagic or sub-benthic fish have lower metal contents than high-trophic-guild carnivores and demersal fish.

**Figure 1:**
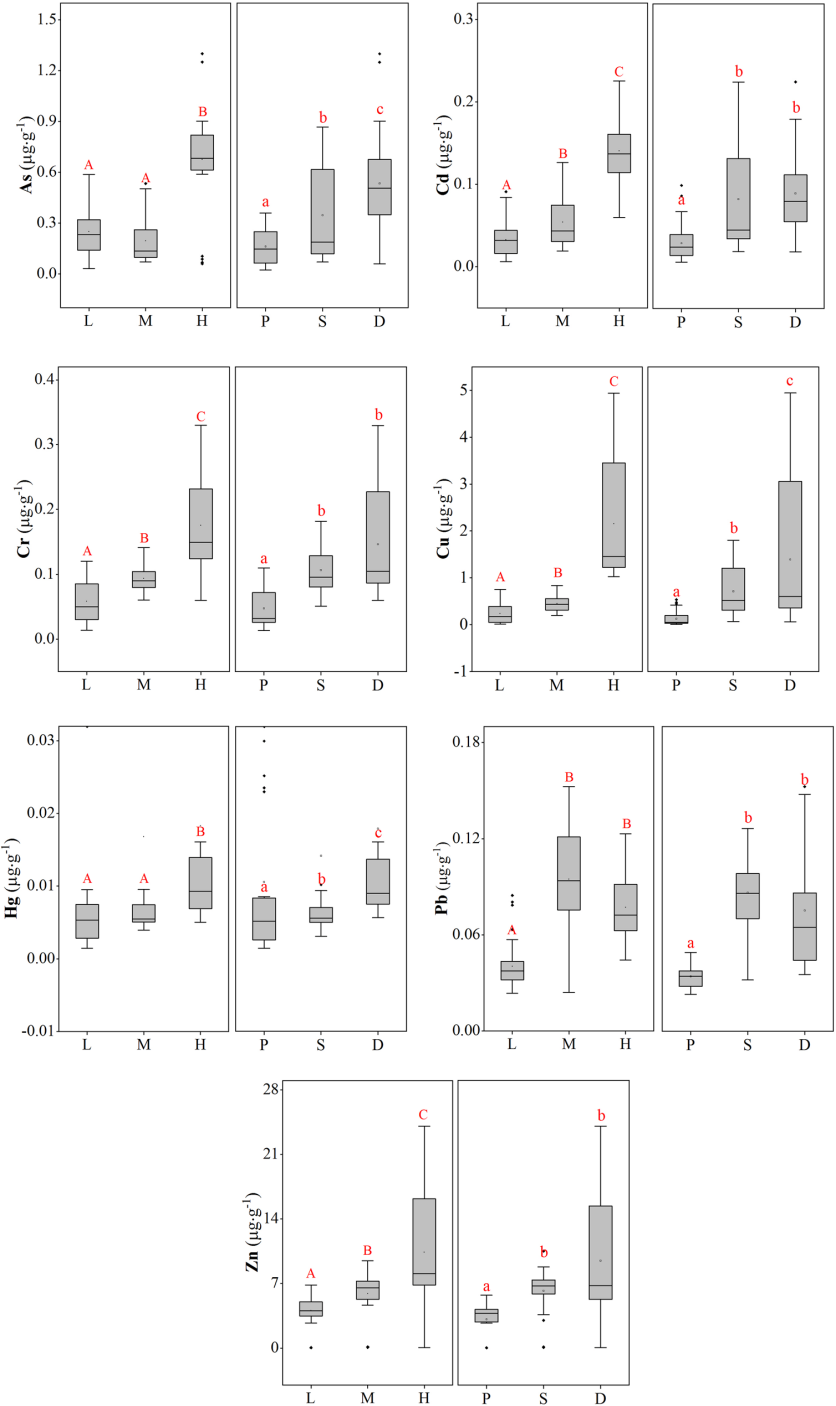
Trace metal contents in fish with different trophic guilds (*L* low-level carnivore, *M* middle-level carnivore, *H* high-level carnivore) and habitat preferences (*P* pelagic, *S* sub-benthic, *D* benthic) from Xincun Lagoon, South China Sea.

Trace metal contents in fish in different trophic levels and in different habitat preferences were compared (Fig. 1). For all metals to be examined, there were significant differences among trophic guilds (p < 0.05). The contents of Cu, Zn, Cd and Cr in high-trophic-guild carnivorous fish were significantly higher than those in other trophic levels (p < 0.05). The high-and-middle-trophic-guild carnivorous fish had higher Pb contents than the low-guild carnivorous fish (p < 0.05). The Hg and As contents in low-or-middle guild carnivorous fish were significantly lower than those in the high-guild carnivorous fish. Similarly, we found that there were significant differences among habitat preferences (p < 0.05). The contents of all trace metals in pelagic fish were significantly lower than those of other habitat preferences (p < 0.05). A significant gradient (p < 0.05) among different habitat preferences was exhibited in the following order: demersal > subdemersal > pelagic for Cu, Hg, As and demersal or subdemersal > pelagic for other metals.

In summary, the fish in high-trophic-guilds or those living at the bottom of water tended to accumulate higher contents of the trace metals, which is consistent with the findings of Bustamante et al.^37^ in Kerguelen Islands and Jiang et al.^38^ in Caizi Lake. This result suggests that the contribution of sediment to the accumulation of trace metals in fish is greater than that of water. Fishes that live in/on sediment mainly feed on benthic organisms, organic debris, or human contaminants (all of which contain high levels of metals), which leads to an accumulation of metal in these species. Thus, for fish species in the same trophic guild, the closer they live to the sediment, the higher the metal content they accumulate. In addition, the high-trophic-guild fish can accumulate metal by preying on the lower-trophic-guild fish, consequently, the high-trophic-guild fish show high metal content, which is consistent with some previous research results^2,38^.

**Figure 2:**
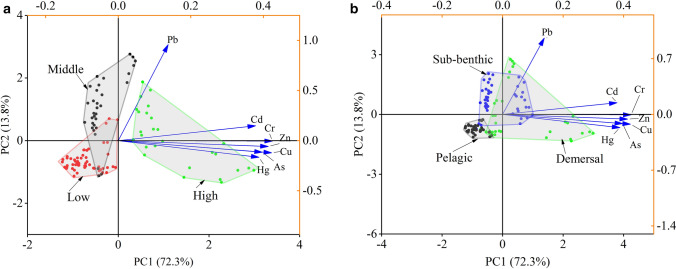
Principal component analysis (PCA) of trace metal contents in the fish with different trophic guilds (**a**) and different habitat preferences (**b**) from Xincun Lagoon, South China Sea.

Principal component analysis (PCA) indicated a significant relationship among trophic levels, habitat preferences and the trace metals found; the first two groups explained 86.1% of the variation in metal contents among the fish species (Fig. 2). The first principal component (PC1) explained 72.3% of the variation and was loaded strongly for Cd, Cr, Zn, Cu, As and Hg, the second principal component (PC2) explained 13.8% of the variation and was loaded strongly for Pb (Fig. 2a). From the trophic level perspective (Fig. 2b), some of the high-trophic-guild carnivorous fish were located on the positive axes of PC1; these were mainly dominated by Cd, Cr, Zn, Cu, As and Hg, while others located on the positive axes of PC2 were dominated by Pb, which was mainly affected by habitat preference. The middle-guild carnivorous fish were mainly located on the positive axes of PC2, which was mainly affected by Pb, and the low-guild carnivorous fish were located on the negative axes of PC1 and PC2. From the habitat preference perspective, some of the demersal fish species were located on the positive axes of PC1, which others were located on the positive axes of PC2. The subbenthic fish were located on the positive axes of PC2, while the pelagic fish were located on the negative axes of PC1 and PC2. These results indicate high species variability in relation to different feeding preferences, the consistent Cd, Cr, Zn, Cu, As and Hg on the PCA indicated homogeneous anthropogenic pollution sources for these six metals. Cabrini^39^ found that fish with different trophic guilds in different regions of Rio de Janeiro in Brazil showed different metal enrichment results, mainly because the metal content of fish food is different in regions with different pollution levels. Therefore, metal accumulation is mainly related to the level of metal content in fish food. In this study, fish samples were collected from a small lagoon with the same degree of pollution and a large range of fish activities, which were basically unaffected by local environmental pollution.

**Figure 3:**
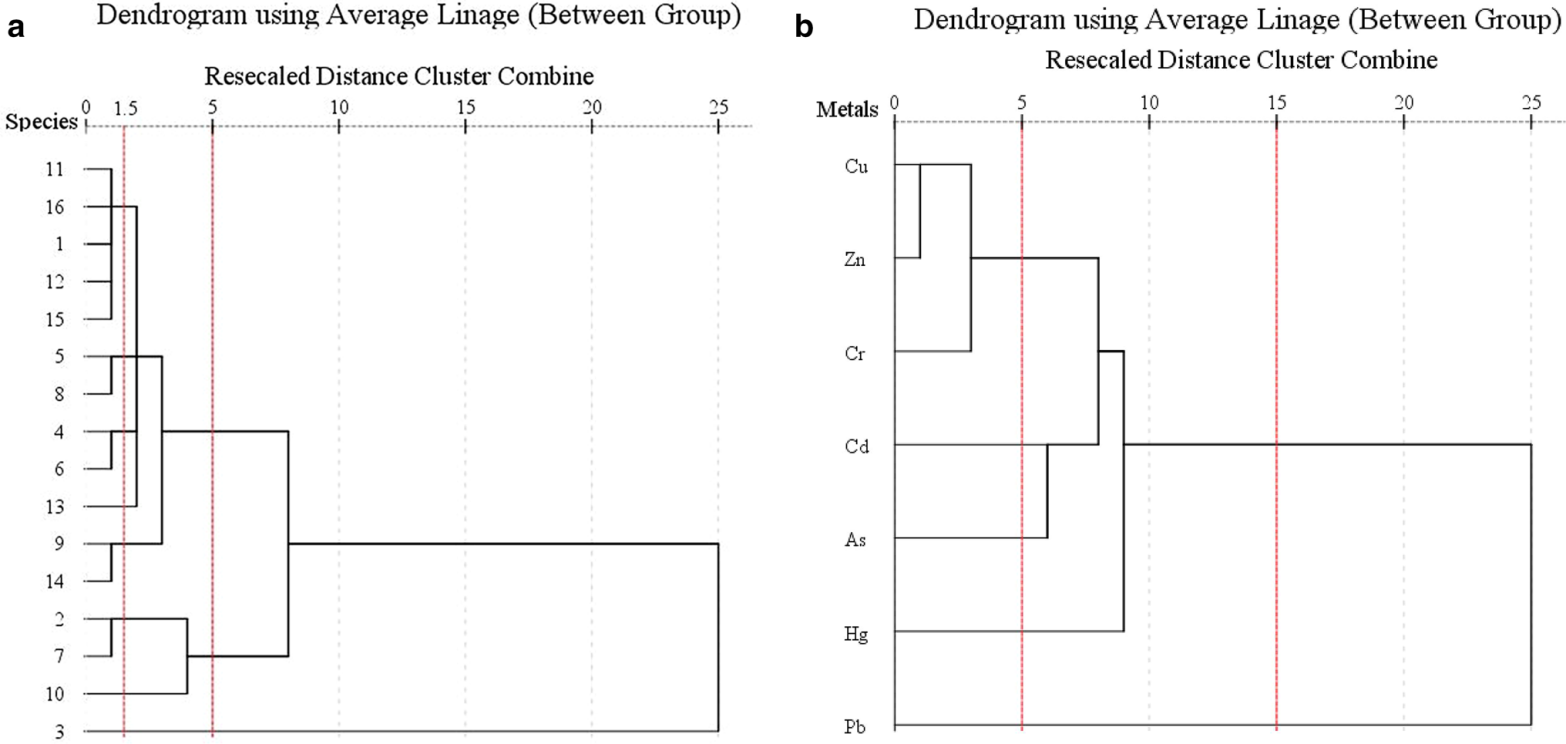
Dendrogram showing species similarity, clustered according to seven trace metals (**a**) and metals homology, cluster according to sixteen fish species (**b**).

Cluster analyses (CA) were used to classify the studied species and metals (Fig. 3). The dendrogram (Fig. 3a) showed that the fish species could be divided into three clusters at a distance of five. Cluster 1 included twelve species (*P. anomala*, *A. sinensis*, *L. brevironstris*, *U. bensasi*, *P. maruadsi*, *G. filamentosus*, *S. chinensis*, *N. gronovii*, *C. dorab*, *B. novae-zeelandiae*, *A. argentatus* and *T. japonicas*) that were primarily from low or middle trophic levels or that lived away from sediment. Cluster 2 included three species (*T. lepturus*, *T. hypargyreus* and *C. sinicus*) that were primarily of high trophic level or demersal preference, while cluster 3 only included *A. sinensis,* which is a high-guild carnivore and with a demersal habitat preference. If cluster 1 was divided further at a distance of 1.5, fish with the same trophic level and habitat preference were grouped into statistically significant clusters. These results further prove that fish with similar ecological characteristics have similar metal enrichment ability. Jiang et al.^38^ also demonstrated a relationship between ecological characteristics and metal contents in fish. In addition, the dendrogram (Fig. 3b) showed that Cu and Zn were grouped into a cluster, the similarity to this cluster decreased in the order: Cr > Cd > As > Hg > Pb. Cu and Zn are micronutrients in living organisms, high contents were found in each fish species. They are regulated by the metabolism and constitute enzymes and hemocyanin, which affect the proper course of many life processes^39,40^. Chromium, which was nearest to the cluster of copper and zinc, has no known biological function but is usually toxic to organisms^41,42^. Cadmium, arsenic, mercury and lead are not related to any biological function and have high toxicity, even at low levels^40,43^.

### Trace metal bioaccumulation

**Figure 4:**
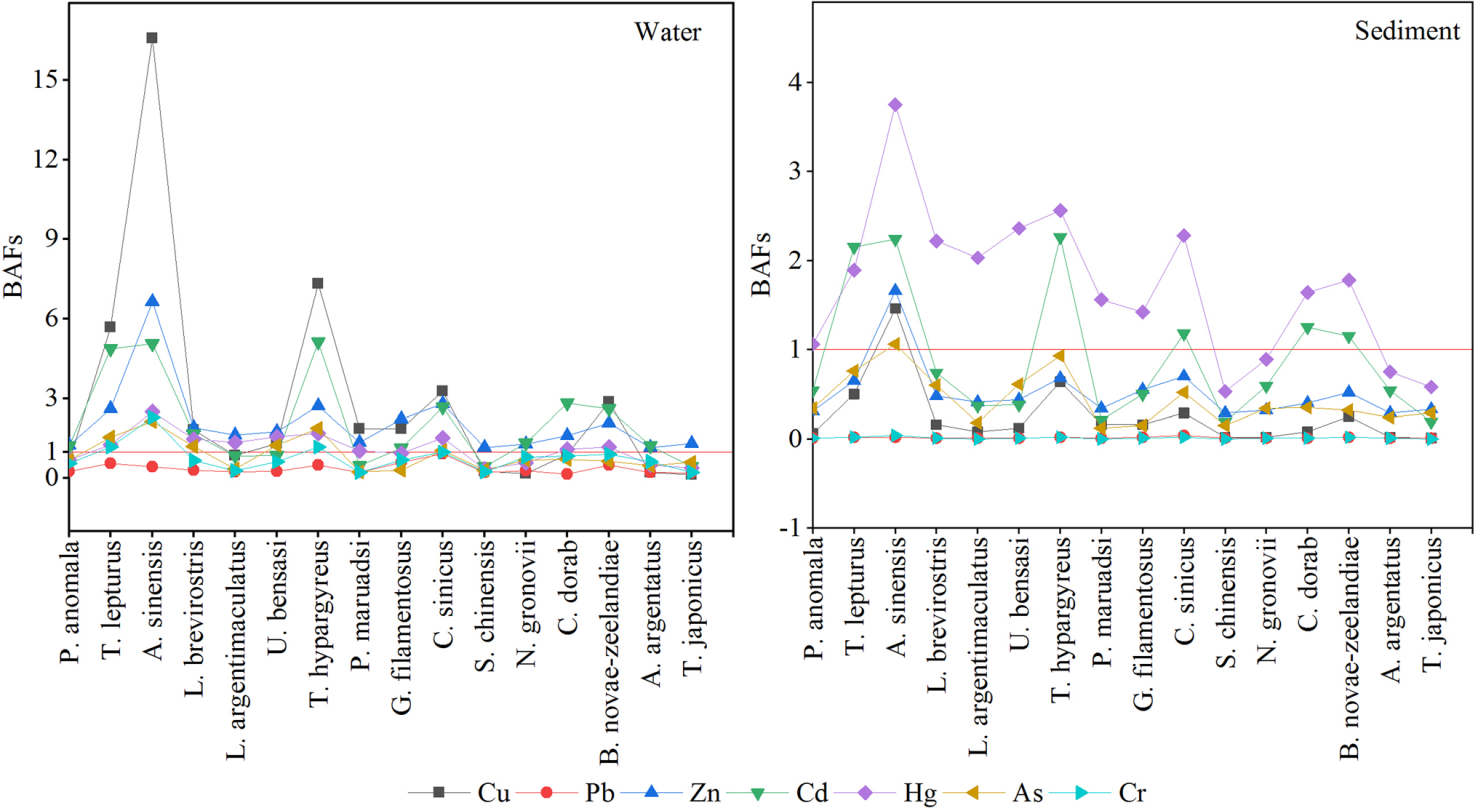
Bioaccumulation factors (BAFs) in the fish samples collected from Xincun Lagoon, South China Sea.

Bioaccumulation factors (BAFs) were calculated as a ratio of trace metal content in fish versus that in water or sediment, which results from the absorption, distribution, and elimination of metal throughout the body of a fish after it has been exposed or fed on a kind of metal element in water, sediment or food; BAFs can determine the tendency of metal elements to accumulate from food or through exposure to an environmental medium^44–46^. Figure 4 shows the BAF values based on the dissolved metal concentrations measured in the water column and metal contents in the surface sediment from Xincun Lagoon. The average BAF values of Cu, Zn, Cd and Hg in water exceeded one, indicating that the bioaccumulation of these metals from water is probable, but it is generally not considered significant unless the BAF is greater than 100^47^. The average BAF values of all study metals in sediments were less than 1 except for Hg and that of Cd which were close to one, indicating that Hg and Cd were more easily accumulated from sediment than other metals. These results suggest that Cd and Hg in the water and sediment of Xincun Lagoon are a potential threat to most fish species living in this water and therefore should be given more attention. Dallinger and Kautzky^48^ suggested that ingestion of metals from food may be a way for fish to accumulate metals. Fish, on the other hand, can ingest organic debris directly from sediment or resuspend it into the water. Therefore, sediment had a higher potential risk than water for fish, especially benthic fish species. However, metal pollution in the water cannot be ignored, because the metal in the water is eventually absorbed by suspended particles and settled into the sediment, which ultimately affects the survival of fish^49,50^.

We found that the fish species in high trophic levels or that live near sediment had higher BAF values (Fig. 4). For instance, BAF peaks in water were positioned in high-guild carnivorous fish (e.g. *T. lepturus*, *A. sinensis* and *T. hypargyreus*) or demersal fish (e.g. *C. sinicus* and *B. novae-zeelandiae*), the BAF values of water for high carnivorous fish were significantly higher than those for benthic fish. This result provides additional evidence that feeding is a major pathway for metal accumulation in fish. The BAFs in sediment showed a similar distribution trend to that of the BAFs of water, but the difference was that the BAFs values of sediment for benthic fish were not as significantly lower as those of the high-level carnivorous fish. This indicated that sediment contributed more to the accumulation of metal in fish than water.

In summary, the results of this study can be used to develop local conservation measures to protect marine ecosystems and guide local fish consumers to eat safely. Future studies should consider the mechanisms of metal accumulation in different fish species.

## Conclusion

This study provided new information on the concentrations of 7 metals (Cu, Pb, Zn, Cd, Hg, As and Cr) in 16 species of fish and their exposed environments (water and sediment) in an important fishing zone: Xincun Lagoon. The contents of all examined metals in the water and sediment were relatively low, and only the contents of Pb and Zn in the water exceeded the threshold, indicating that the potential ecological risks were slight. The metal contents in fish tissues were also relatively low, the metal contents of species were significantly different among trophic levels and habitat preferences. Our results suggested that the contents of accumulated metals in fish were affected by both habitat preference and trophic level and the latter was more significant. Furthermore, the fish in the same trophic levels or with similar habitat preferences had homogeneous sources of metal. The BAF results demonstrated that the Cd and Hg in the water and sediment of Xincun Lagoon are potential threats for most fish species living in this water, sediment contributed more to the accumulation of metal in fish than water. Our results can provide valuable information for understanding metal pollution in economic fish and the accumulation patterns of metals in Xincun Lagoon.

## Materials and methods

### Study area

**Figure 5:**
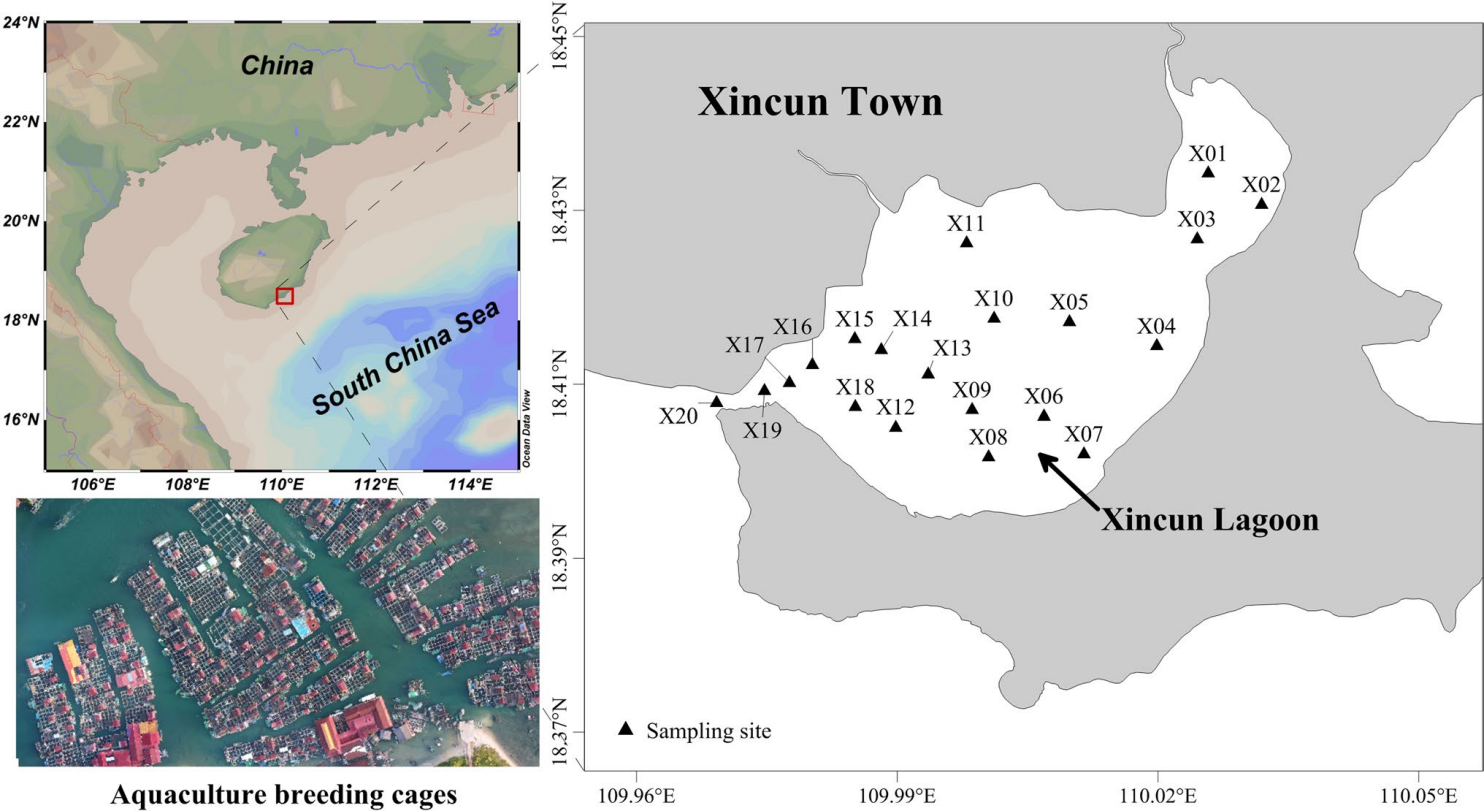
Map of study area and sampling sites in Xincun Lagoon, South China Sea. (Software: Ocean Data View 4. The map was obtained from the ODV software’s own map resources, Schlitzer, Reiner, Ocean Data View, odv.awi.de, 2020).

Xincun Lagoon is a nearly closed natural fishing port completely controlled by the tide in southern Hainan Island, China (Fig. 5). It is located between the latitudes of 18°24′ and 18°25′ N and the longitudes of 109°57′ and 110°00′ E, with an area of approximately 22 km^2^ and a narrow mouth entrance of approximately 150 m, the lagoon is an important mariculture area in China. Currently, there are more than 700 seawater breeding cages (approximately 15% of the entire lagoon area) and 500 fishing boats in the lagoon, with hundreds of thousands of "Danjia" people living on the water. The contaminants from aquaculture, sewage and shipping have caused serious pollution of the environment in the lagoon^30^. According to local fishermen and field investigations, the disease and death rate of farmed fish in the lagoon has increased continuously in recent years, resulting in a significant decline in the yield and quality of fish products. The ecological environment and the issues surrounding Xincun Lagoon has attracted the attention of both scholars and managers.

### Sampling and sample pretreatment

Water, sediment and fish samples were collected from 20 sampling sites in the Xincun Lagoon during November, 2016 (Fig. 5). Duplicate of 250 mL water samples were taken into polyethylene bottles at 0.5 m below the surface water using a Niskin water sampler (2.5 L, GENERAL OCEANIC, USA). The water samples were immediately filtered through a vacuum filter (CKY, China) with 0.45 µm Millipore membrane and acidified with nitric acid (Guaranteed reagent (GR), MACKLIN, China) to pH < 2 until analyzed. Sediment samples 0.5 kg surface layer (0–5 cm) were collected into polyethylene bags using a bottom sampler. The sediment samples were dried in an oven at 80 °C, ground into powder by a manual agate mortar (LC, China), sifted by a 96 µm nylon sieve and stored until digest. The fish samples were caught by local fishermen. A total of 110 samples of sixteen types of common local economic fish species were collected. All collected samples were classified, weighed, measured for length and dissected, their ecological characteristics are listed in Table 2. The muscular tissues of fish samples were dried, ground into powder, sifted and stored until digest in the same way as sediment samples.

**Table 2:** The ecological characteristics and recorded morphometric measures of sixteen fish species collected from Xincun Lagoon, South China Sea.

Trophic guild	Habitat	Scientific name	Family	No. of samples	Length (cm)	Weight (g)
Low carnivore	Pelagic	*Lutjanus argentimaculatus*	*Lutjanus*	5	12 ± 2.2	43.7 ± 12.9
		*Pecapterus maruadsi*	*Caranx*	8	19.1 ± 3.1	73.4 ± 11.3
		*Stolephorus chinensis*	*Stolephorus*	3	11.0 ± 1.0	21.9 ± 3.3
		*Nomeus gronovii*	*Nomeus*	6	15.6 ± 3.6	338.7 ± 61.2
	Sub-benthic	*Argyrosomus argentatus*	*Argyrosomus*	4	14.1 ± 2.5	49.9 ± 6.3
		*Trachurus japonicus*	*Trachurus*	7	18.1 ± 1.6	35.8 ± 5.8
		*Psenopsis anomala*	*Psenopsis*	3	13.8 ± 1.5	68.1 ± 19.6
	Demersal	*Leiognathus brevirostris*	*Leiognathus*	7	8.7 ± 1.3	21.6 ± 4.1
		*Upeneus bensasi*	*Upeneus*	3	11.6 ± 1.2	37.1 ± 8.4
		*Brachypleura novae-zeelandiae*	*Brachypleura*	4	12.7 ± 1.2	23.9 ± 4.1
Middle carnivore	Pelagic	*Chirocentrus dorab*	*Chirocentrus*	3	30.4 ± 8.2	386.1 ± 47.9
	Sub-benthic	*Gerres filamentosus*	*Gerres*	23	17.8 ± 1.1	112.0 ± 15.4
	Demersal	*Cynoglossus sinicus*	*Cynoglossus*	6	15.3 ± 1.6	30.4 ± 4.6
High carnivore	Sub-benthic	*Trichiurus lepturus*	*Tentoriceps*	14	43.6 ± 11.4	297.2 ± 58.2
		*Thamnaconus hypargyreus*	*Thamnaconus*	4	10.0 ± 1.5	48.4 ± 24.4
	Demersal	*Acipenser sinensis*	*Acipenser*	10	37.1 ± 5.4	447.9 ± 45.3

### Determination of trace metals

The determination of Cu, Pb, Zn, Cd and Cr in the water samples was based on the chelation of the metals with sodium diethyldithiocarbamate (DDTC, MACKLIN, China) and ammonium pyrrolidine dithiocarbamate (APDC, MACKLIN, China) and the extraction in which chelation was then extracted into methyl isobutyl ketone (MIBK, MACKLIN, China). Briefly, the pH of the 20 mL filtered water sample was adjusted to the appropriate value with ammonia and hydrochloric acid (pH = 5 ~ 6 for Cu, Pb and Cd; pH = 3.8 ± 0.2 for Zn and Cr). 1 mL of ammonium acetate buffer solution (pH = 5 for Cu, Pb and Cd; pH = 3.8 for Zn and Cr) and 1 mL of mixed solution of 10 g·L^-1^ DDTC and APDC were added, then 2.5 mL MIBK was added. The mixture was shaken for 2 min and allowed to stand for 20 min so that the organic phase could separate. The organic phase was determined using atomic absorption spectrometer (AAS 900 T, PE, US). For the determination of Hg and As in the water samples, 25 mL of the water sample were added to the glass tubes, then 2 mL of potassium borohydride reductant was mixed with it, and the mercury vapor (for Hg)/hydrogen arsenide (for As) formed were determined using atomic fluorescence spectrometer (AFS XGY-1011A, China) under the action of carrier gas N_2_. Detailed operating procedures are described in the Specification for Marine Monitoring of China (SMMC) (GB17378.4-2007, ICS 07.060 A 45)^51^.

The digestion and determination of metals in the sediment samples were performed according to SMMC. Briefly, Teflon digestion vessels with 0.2 g of dry sediment samples were added to 6 mL nitric acid, 2 mL perchloric acid (GR, SCR, China), 1 mL hydrochloric acid (GR, SCR, China) and left for 24 h for predigestion. Then the digestion vessel was transferred to the microwave digestion apparatus (ANTON PAAR, Austria) for digestion according to a four-step program (10 min at 180 °C with 800 W, 10 min at 190 °C with 900 W and as a last step 10 min at 100 °C with 400 W). After digestion, the vessel was heated at 80 °C on a heater until the remaining acid to approximately 1 mL. The remaining solution was transferred to a polyethylene tube (LABSBUY, China) and diluted to 25 mL with Milli-Q water (MILLIPORE, US). The diluted solutions were filtered by using syringe filter (SFPTFE013022SL-13, pore size = 45 µm, ASONE, Japan) and stored in 25 mL polyethylene tube. Cu, Pb, Zn, Cd and Cr of the digested solutions were determined by AAS, while Hg and As were determined by AFS.

The digestion and determination of metals in the fish samples were carried out following the steps described by SMMC. Dried fish samples were digested with 9 mL nitric acid and 2 mL hydrogen peroxide (GR, MACKLIN, China) following in the same digestion steps as the sediment samples.

### Quality control and assurance

Each batch of experiments ran simultaneously with blank, parallel samples and certified reference materials (CRMs), to eliminate any systematic errors. The CRMs involved in this study included seawater reference solutions (GBW(E) 080040 for Cu, Pb, Zn, Cd, Cr; GBW(E) 080042 for Hg; GBW(E)080230 for As; BZWZ, China), offshore marine sediments (GBW07314; Research Center for Eco-Environmental Sciences, Chinese Academy of Sciences) and *Undaria pinnatifida* (GBW(E)100395; Research Center for Eco-Environmental Sciences, Chinese Academy of Sciences). All data results were validated against certified reference materials which provided recovery rates between 82 and 108% see Supplementary Table 1. All results were in good agreement with the certified values. The trace metal concentrations were expressed as µg·g^−1^ in sediment and fish and recorded in water as µg·L^−1^, the mean conversion factor used to convert from dry weight to wet weight was 0.2 for fish samples.

### Statistical analysis

The differences in trace metal contents among fish species in different trophic levels and different habitat preferences were analyzed using the statistical analysis software IBM-SPSS 24.0. If the data analyzed were normally distributed and had equal variance, the difference was assessed using a one-way ANOVA, followed by *Tukey*’s post hoc tests for pairwise comparisons if significant differences (*p* < 0.05) were found. The differences were analyzed with a nonparametric *Kruskal–Wallis H* test if abnormality or unequal variance was found, followed by *Dunn’s* tests with adjusted *p* values for pairwise comparisons.

Principal component analysis (PCA) was used to determine the difference among species in various trophic levels and with various habitat preferences based on the metal data. In all cases, the data were standardized by using the Z-score transformation. The PCA was performed using Origin 2018 software with PCA-APP (OriginLab, Corp., US).

### Ethical approval

All methods used during this study were carried out in accordance with relevant guidelines and regulations. All experimental protocols were approved by the Second Institute of Oceanography, MNR. The study was approved by the ethic committee of the Second Institute of Oceanography, MNR.

## Supplementary information

Supplementary Information

## References

[1] Mason RP (2013). Trace Metals in Aquatic Systems.

[2] Chouvelon T (2019). Patterns of trace metal bioaccumulation and trophic transfer in a phytoplankton-zooplankton-small pelagic fish marine food web. Mar. Pollut. Bull..

[3] Lewis SL, Maslin MA (2015). Defining the anthropocene. Nature.

[4] Terra BF, Araújo FG, Calza CF, Lopes RT, Teixeira TP (2007). Heavy metal in tissues of three fish species from different trophic levels in a tropical BRAZILIAN river. Water Air Soil Poll..

[5] Eisler, R. Zink Hazards to fish, wildlife and invertebrates: A synoptic review, 99 (1988).

[6] Clark R, Frid C, Attrill M (1997). Marine Pollution.

[7] Asuquo FE, Ewa-Oboho I, Asuquo EF, Udo PJ (2004). Fish species used as biomarker for heavy metal and hydrocarbon contamination for cross River Nigeria. Environmentalist.

[8] Vu CT, Lin C, Yeh G, Villanueva MC (2017). Bioaccumulation and potential sources of heavy metal contamination in fish species in Taiwan: Assessment and possible human health implications. Environ. Sci. Pollut. Res. Int..

[9] Alipour H, Pourkhabbaz A, Hassanpour M (2014). Estimation of potential health risks for some metallic elements by consumption of fish. Water Qual. Expos. Hea..

[10] Kambe T, Nishito Y, Fukue K, Collins JF (2017). Chapter 23—Zinc transporters in health and disease A2. Molecular, Genetic, and Nutritional Aspects of Major and Trace Minerals.

[11] Merian, E. Metals and Their Compounds in the Environments, (VCH, 1991).

[12] Vos G, Lammers H, van Delft W (1988). Arsenic, cadmium, lead and mercury in meat, livers and kidneys of sheep slaughtered in The Netherlands. Z. Lebensm Unters Forsch.

[13] Pilehvarian AA, Malekirad AA, Bolandnazar N-S, Rezaei M (2016). Heavy metal bioaccumulation in different fish species in the coast of the Persian Gulf, Iran. Toxin. Rev..

[14] Telisman S, Colak B, Pizent A, Jurasovic J, Cvitkovic P (2007). Reproductive toxicity of low-level lead exposure in men. Environ. Res..

[15] Kapaj S, Peterson H, Liber K, Bhattacharya P (2006). Human health effects from chronic arsenic poisoning—a review. J. Environ. Sci. Heal..

[16] Perugini M (2016). Effect of cooking on total mercury content in Norway lobster and European hake and public health impact. Mar. Pollut. Bull..

[17] Rahman MS, Saha N, Molla AH (2013). Potential ecological risk assessment of heavy metal contamination in sediment and water body around Dhaka export processing zone Bangladesh. Environ. Earth Sci..

[18] Bervoets L, Blust R (2003). Metal concentrations in water, sediment and gudgeon (Gobio gobio) from a pollution gradient: Relationship with fish condition factor. Environ. Pollut..

[19] Idriss AA, Ahmad AK (2015). Heavy metal concentrations in fishes from Juru River, estimation of the health risk. B. Environ. Contam. Tox..

[20] Fallah AA, Saei-Dehkordi SS, Nematollahi A, Jafari T (2011). Comparative study of heavy metal and trace element accumulation in edible tissues of farmed and wild rainbow trout (*Oncorhynchus mykiss*) using ICP-OES technique. Microchem. J..

[21] Copat C (2013). Heavy metals concentrations in fish and shellfish from eastern Mediterranean Sea: Consumption advisories. Food Chem. Toxicol..

[22] Miri M (2017). Health risk assessment of heavy metal intake due to fish consumption in the Sistan region, Iran. Environ. Monit. Assess..

[23] Varol M, Kaya GK, Alp SA, Sunbul MR (2018). Trace metal levels in rainbow trout (*Oncorhynchus mykiss*) cultured in net cages in a reservoir and evaluation of human health risks from consumption. Biol. Trace Elem. Res..

[24] Kalyoncu L, Kalyoncu H, Arslan G (2011). Determination of heavy metals and metals levels in five fish species from Işikli Dam Lake and Karacaören Dam Lake (Turkey). Environ. Monit. Assess..

[25] Li P (2015). Heavy metal bioaccumulation and health hazard assessment for three fish species from Nansi Lake China. B. Environ. Contam. Tox..

[26] Roesijiadi G, Robinson WE, Malins DC, Ostrander GG (1994). Metal regulation in aquatic animals: mechanism of uptake, accumulation and release. Aquatic Toxicology (Molecular Biochemical and Cellular Perspectives).

[27] Kalay M, Ay O, Canli M (1999). Heavy metal concentrations in fish tissues from the northeast Mediterranean Sea. Bull. Environ. Contam. Toxicol..

[28] Kalay M, Canli M (2000). Elimination of essential (Cu, Zn) and non-essential (Cd, Pb) metals from tissues of a freshwater fish Tilapia zilli. Turk. J. Zool..

[29] Rainbow PS (2002). Trace metal concentrations in aquatic invertebrates: Why and so what?. Environ. Pollut..

[30] Yang H, Wang W, Li Q, Fu Q (2015). Discussion on the development of deep-water cage culture in Lingshui County, Hainan. Ocean Dev. Manag..

[31] Liu F (2016). Role of zinc content on corrosion performance for cold galvanized coatings. Corros. Sci. Protect. Technol..

[32] Xu J, Xia Q, Zhang F (2018). A study of lead corrosion of diesel engine oil. China Pet. Process..

[33] Thuy HTT, Tobschall HJ, An PV (2000). Trace element distributions in aquatic sediments of Danang–Hoian area Vietnam. Environ. Geol..

[34] Yang Y (2016). Grain size distribution of surface sediments and sedimentary environment in the lagoon of Xincun Hainan Island. Haiyang Xuebao.

[35] EC. Official Journal of the European Union Legislation, 364, (2006).

[36] CNS. China National Standard, (2017).

[37] Bustamante P, Bocher P, Cherel Y, Miramand P, Caurant F (2003). Distribution of trace elements in the tissues of benthic and pelagic fish from the Kerguelen Islands. Sci. Total Environ..

[38] Jiang Z (2018). Metal concentrations and risk assessment in water, sediment and economic fish species with various habitat preferences and trophic guilds from Lake Caizi, Southeast China. Ecotoxicol. Environ. Saf..

[39] Cabrini TMB (2018). Investigating heavy metal bioaccumulation by macrofauna species from different feeding guilds from sandy beaches in Rio de Janeiro, Brazil. Ecotox. Environ. Safe..

[40] Jakimska A, Konieczka P, Skora K, Namiesnik J (2011). Bioaccumulation of metals in tissues of marine animals, part II: Metal concentrations in animal tissues. Pol. J. Environ. Stud..

[41] Ochiai EL (1995). Toxicity of heavy metals and biological defense: principles and applications in bioinorganic chemistry-VII. J. Chem. Educ..

[42] Natale GS, Basso NG, Ronco AE (2000). Effect of Cr(VI) on early life stages of three species of hylid frogs (Amphibia, Anura) from South America. Environ. Toxicol..

[43] Chiarelli R, Roccheri MC (2014). Marine invertebrates as bioindicators of heavy metal pollution. Open J. Metal.

[44] Lau S, Mohamed M, Tan Chi Yen A, Suut S (1998). Accumulation of heavy metals in freshwater molluscs. Sci. Total Environ..

[45] Subotic S (2013). Heavy metal and trace element bioaccumulation in target tissues of four edible fish species from the Danube River (Serbia). Ecotoxicol. Environ. Saf..

[46] Voigt CL (2015). Bioconcentration and bioaccumulation of metal in freshwater Neotropical fish Geophagus brasiliensis. Environ. Sci. Pollut. Res. Int..

[47] Hao Z (2019). Heavy metal distribution and bioaccumulation ability in marine organisms from coastal regions of Hainan and Zhoushan, China. Chemosphere.

[48] Dallinger R, Kautzky H (1985). The importance of contaminated food for the uptake of heavy metals by rainbow trout (Salmo gairdneri): A field study. Oecologia.

[49] Gambrell RP, Wiesepape JB, Patrick WH, Duff MC (1991). The effects of pH, redox, and salinity on metal release from a contaminated sediment. Water, Air, Soil Poll..

[50] Riedel GF, Sanders JG, Osman RW (1999). Biogeochemical control on the flux of trace elements from estuarine sediments: Effects of seasonal and short-term hypoxia. Mar. Environ. Res..

[51] SOA. in (GB 17378–2007) (Standardization Administration of the People's Republic of China (SAC), Beijing, 2007).

