# Supplementary material for: Species-specific bioaccumulation of trace metals among fish species from Xincun Lagoon, South China Sea
Source: Sci Rep. 2020 Dec 11;10:21800. doi: 10.1038/s41598-020-77917-y (PMC7732978; doi:10.1038/s41598-020-77917-y)
Supplement: Supplementary file 1 — Supplementary Information [file 41598_2020_77917_MOESM1_ESM.docx]

**Supplementary Table 1**

The average recoveries of trace metals in the CRMs (Seawater Reference Solutions: GBW(E) 080040 for Cu, Pb, Zn, Cd and Cr, GBW(E) 080042 for Hg, GBW(E)080230 for As, (BZWZ, China); Offshore Marine Sediments (GBW07314; Research Center for Eco-Environmental Sciences, Chinese Academy of Sciences); *Undaria pinnatifida* (GBW(E)100395; Research Center for Eco-Environmental Sciences, Chinese Academy of Sciences))

| **Metals** | **Water** | | | **Sediment** | | | **Organism** | | |
| --- | --- | --- | --- | --- | --- | --- | --- | --- | --- |
|  | ***Cv***  **(µg·L^-1^)** | ***Mv***  **(µg·L^-1^)** | ***aR***  **(%)** | ***Cv***  **(µg·g^-1^)** | ***Mv***  **(µg·g^-1^)** | ***aR***  **(%)** | ***Cv***  **(µg·g^-1^)** | ***Mv***  **(µg·g^-1^)** | *aR*  (%) |
| Cu | 5 ± 0.4 | 4.8 ± 0.2 | 95 | 28 ± 2 | 28.2 ± 1.2 | 101 | 30.9 ± 1.3 | 30.7 ± 0.6 | 99 |
| Pb | 10 ± 0.6 | 9.48 ± 1.50 | 95 | 23 ± 4 | 22.2 ± 1.2 | 97 | 0.53 ± 0.08 | 0.47 ± 0.01 | 88 |
| Zn | 70 ± 3 | 72.1 ± 1.7 | 103 | 77 ± 6 | 82.1 ± 2.7 | 107 | 67.7 ± 3.4 | 69.5 ± 1.4 | 103 |
| Cd | 1 ± 0.06 | 0.94 ± 0.02 | 94 | 0.17 ± 0.04 | 0.16 ± 0.01 | 92 | 1.79 ± 0.14 | 1.73 ± 0.03 | 96 |
| Hg | 1 ± 0.06 | 1.035 ± 0.167 | 104 | 0.048 ± 0.012 | 0.044 ± 0.003 | 92 | 0.017 ± 0.004 | 0.014 ± 0.001 | 82 |
| As | 100 ± 4 | 95.5 ± 1.6 | 96 | 10.3 ± 1.4 | 9.63 ± 0.48 | 93 | 43.7 ± 3.9 | 45.4 ± 2.0 | 104 |
| Cr | 5 ± 0.4 | 4.8 ± 0.2 | 97 | 46 ± 8 | 49.6 ± 1.95 | 108 | 0.52 ± 0.04 | 0.54 ± 0.02 | 103 |

*Cv*: Certified Value; *Mv*: Measured Value; *aR*: Average Recovery.

**Supplementary Table 2**

Depth (m), Temperature (^o^C), Salinity, pH, trace metal concentrations (µg·L^-1^, mean ± SD) and their legislation thresholds (LT) in the water of Xincun Lagoon, South China Sea.

| **Sites** | **Depth**  **(m)** | **Temperature**  **(^o^C)** | **Salinity** | **pH** | **Cu**  **(µg·L^-1^)** | **Pb**  **(µg·L^-1^)** | **Zn**  **(µg·L^-1^)** | **Cd**  **(µg·L^-1^)** | **Hg**  **(µg·L^-1^)** | **As**  **(µg·L^-1^)** | **Cr**  **(µg·L^-1^)** |
| --- | --- | --- | --- | --- | --- | --- | --- | --- | --- | --- | --- |
| X01 | 1.0 | 25.0 | 21.9 | 7.98 | 1.8 ± 0.1 | **1.73 ± 0.05** | **21.0 ± 1.1** | 0.31 ± 0.03 | 0.029 ± 0.001 | 3.2 ± 0.1 | 0.4 ± 0.0 |
| X02 | 2.0 | 25.2 | 29.3 | 7.96 | 1.4 ± 0.1 | **1.37 ± 0.07** | **22.4 ± 0.6** | 0.34 ± 0.03 | 0.025 ± 0.002 | 2.4 ± 0.2 | 0.8 ± 0.1 |
| X03 | 3.5 | 27.9 | 30.2 | 8.03 | 1.3 ± 0.2 | **1.16 ± 0.03** | **23.2 ± 0.9** | 0.37 ± 0.01 | 0.020 ± 0.000 | 1.6 ± 0.1 | 1.2 ± 0.1 |
| X04 | 7.0 | 28.0 | 30.5 | 8.02 | 1.0 ± 0.1 | 0.86 ± 0.04 | 19.5 ± 0.7 | 0.37 ± 0.04 | 0.020 ± 0.001 | 1.8 ± 0.1 | 1.4 ± 0.2 |
| X05 | 7.0 | 27.9 | 30.3 | 8.03 | 0.8 ± 0.3 | 0.20 ± 0.02 | **48.6 ± 1.9** | 0.29 ± 0.01 | 0.020 ± 0.001 | 1.8 ± 0.3 | 1.2 ± 0.1 |
| X06 | 8.0 | 28.8 | 31.0 | 8.17 | 1.2 ± 0.1 | 0.48 ± 0.02 | **23.3 ± 0.4** | 0.40 ± 0.02 | 0.023 ± 0.001 | 1.7 ± 0.1 | 1.1 ± 0.1 |
| X07 | 9.0 | 27.9 | 30.0 | 8.03 | 0.8 ± 0.2 | 0.83 ± 0.05 | 13.4 ± 0.5 | 0.32 ± 0.01 | 0.020 ± 0.002 | 3.6 ± 0.2 | 1.3 ± 0.1 |
| X08 | 8.0 | 27.7 | 29.6 | 8.04 | 0.9 ± 0.2 | 0.83 ± 0.04 | **21.3 ± 1.0** | 0.30 ± 0.01 | 0.021 ± 0.001 | 1.3 ± 0.1 | 1.1 ± 0.1 |
| X09 | 9.0 | 28.8 | 30.8 | 8.17 | 1.1 ± 0.2 | 0.93 ± 0.07 | **33.5 ± 0.8** | 0.40 ± 0.02 | 0.029 ± 0.001 | 1.9 ± 0.1 | 0.5 ± 0.0 |
| X10 | 8.0 | 28.0 | 30.2 | 8.01 | 1.0 ± 0.1 | 0.99 ± 0.06 | **32.1 ± 0.6** | 0.37 ± 0.03 | 0.025 ± 0.001 | 1.9 ± 0.1 | 0.8 ± 0.1 |
| X11 | 9.0 | 27.8 | 30.3 | 8.02 | 1.1 ± 0.2 | 0.60 ± 0.03 | **32.4 ± 0.3** | 0.35 ± 0.01 | 0.023 ± 0.001 | 1.5 ± 0.1 | 1.1 ± 0.1 |
| X12 | 9.5 | 28.6 | 30.9 | 8.16 | 1.0 ± 0.3 | 0.89 ± 0.04 | **62.7 ± 1.1** | 0.32 ± 0.01 | 0.026 ± 0.002 | 1.9 ± 0.0 | 0.7 ± 0.1 |
| X13 | 9.0 | 28.1 | 28.2 | 7.98 | 0.9 ± 0.1 | 0.26 ± 0.01 | **36.9 ± 0.9** | 0.31 ± 0.03 | 0.024 ± 0.001 | 2.9 ± 0.2 | 0.3 ± 0.1 |
| X14 | 6.0 | 28.0 | 31.1 | 8.00 | 1.3 ± 0.2 | 0.97 ± 0.07 | **26.0 ± 0.3** | 0.35 ± 0.02 | 0.032 ± 0.001 | 3.5 ± 0.1 | 0.6 ± 0.1 |
| X15 | 8.0 | 28.4 | 31.7 | 8.17 | 1.5 ± 0.1 | **1.43 ± 0.06** | **20.2 ± 0.5** | 0.32 ± 0.02 | 0.039 ± 0.002 | 1.7 ± 0.0 | 0.5 ±0.1 |
| X16 | 8.0 | 28.8 | 32.3 | 8.20 | 0.8 ± 0.1 | 0.89 ± 0.04 | 18.6 ± 0.4 | 0.36 ± 0.01 | 0.035 ± 0.002 | 1.2 ± 0.1 | 0.3 ± 0.1 |
| X17 | 8.0 | 28.3 | 33.0 | 8.06 | 0.6 ± 0.2 | 0.62 ± 0.01 | 12.6 ± 0.1 | 0.43 ± 0.03 | 0.030 ± 0.001 | 1.6 ± 0.1 | 1.3 ± 0.2 |
| X18 | 3.0 | 28.2 | 29.3 | 8.00 | 0.6 ± 0.1 | 0.07 ± 0.01 | **23.3 ± 0.7** | 0.60 ± 0.04 | 0.027 ± 0.001 | 3.1 ± 0.2 | 0.4 ± 0.0 |
| X19 | 12.0 | 28.5 | 32.3 | 8.23 | 0.9 ± 0.2 | 0.18 ± 0.01 | **24.1 ± 0.3** | 0.49 ± 0.02 | 0.033 ± 0.002 | 2.4 ± 0.1 | 0.6 ± 0.1 |
| X20 | 2.0 | 28.7 | 31.9 | 8.15 | 1.3 ± 0.2 | 0.42 ± 0.02 | **25.8 ± 0.8** | 0.36 ± 0.01 | 0.039 ± 0.002 | 1.4 ± 0.1 | 0.7 ± 0.1 |
| LT | SQSC^1^ | | | | 5.0 | 1.0 | 20.0 | 1.0 | 0.05 | 20.0 | 50.0 |
|  | USEPA^2^ | | | | 3.1 | 8.1 | 81.0 | 8.8 | 0.94 | 36.0 | - |

^1^ The primary standard values in the Seawater Quality Standard of China (GB 3097-1997);

^2^ Criteria continuous concentration (CCC) in national recommended water quality criteria of the United States (USEPA-2009).

**Supplementary Table 3**

Trace metal concentrations (µg·g^-1^, mean ± SD) and their legislation thresholds in the sediment of Xincun Lagoon, South China Sea.

| **Sites** | **Cu** | **Pb** | **Zn** | **Cd** | **Hg** | **As** | **Cr** |
| --- | --- | --- | --- | --- | --- | --- | --- |
| X01 | 2.1 ± 0.1 | 6.8 ± 0.2 | 10.8 ± 0.7 | 0.07 ± 0.01 | 0.004 ± 0.001 | 2.19 ± 0.31 | 2.1 ± 0.2 |
| X03 | 15.3 ± 0.3 | 16.3 ± 0.4 | 57.7 ± 1.1 | 0.42 ± 0.03 | 0.003 ± 0.001 | 3.00 ± 0.16 | 32.0 ± 1.1 |
| X05 | 11.1 ± 0.2 | 10.6 ± 0.1 | 54.4 ± 1.0 | 0.34 ± 0.01 | 0.015 ± 0.002 | 3.51 ± 0.29 | 32.3 ± 1.5 |
| X06 | 22.6 ± 0.5 | 31.4 ± 0.6 | 85.3 ± 0.8 | **0.70 ± 0.02** | 0.053 ± 0.003 | 5.23 ± 0.30 | 67.0 ± 1.4 |
| X07 | 5.6 ± 0.2 | 11.8 ± 0.3 | 37.7 ± 0.5 | 0.18 ± 0.01 | 0.005 ± 0.001 | 4.37 ± 0.13 | 12.1 ± 0.8 |
| X09 | 25.3 ± 0.6 | 32.2 ± 0.8 | 96.1 ± 1.1 | **0.70 ± 0.01** | 0.059 ± 0.002 | 5.43 ± 0.27 | 67.0 ± 1.1 |
| X11 | 28.5 ± 0.4 | 29.3 ± 0.5 | 94.2 ± 1.3 | **0.62 ± 0.02** | 0.008 ± 0.001 | 4.24 ± 0.18 | 75.7 ± 0.9 |
| X12 | 22.5 ± 0.6 | 29.4 ± 0.1 | 99.1 ± 0.8 | **0.66 ± 0.04** | 0.048 ± 0.002 | 6.09 ± 0.09 | 72.1 ± 0.6 |
| X14 | 3.5 ± 0.1 | 11.5 ± 0.4 | 28.5 ± 0.3 | 0.10 ± 0.01 | 0.008 ± 0.001 | 5.41 ± 0.12 | 6.7 ± 0.3 |
| X15 | 3.5 ± 0.1 | 12.0 ± 0.3 | 57.3 ± 0.7 | 0.12 ± 0.02 | 0.003 ± 0.001 | 3.68 ± 0.13 | 6.2 ± 0.2 |
| X18 | 1.4 ± 0.1 | 7.1 ± 0.1 | 11.3 ± 0.2 | 0.04 ± 0.01 | 0.002 ± 0.000 | 1.48 ± 0.07 | 4.1 ± 0.1 |
| X20 | 8.9 ± 0.1 | 26.2 ± 0.6 | 69.3 ± 0.7 | 0.15 ± 0.01 | 0.012 ± 0.001 | 6.33 ± 0.22 | 54.0 ± 0.7 |
| MSQSC^3^ | 35.0 | 60.0 | 150.0 | 0.50 | 0.200 | 20.00 | 80.0 |
| CSQG^4^ | 108.0 | 112.0 | 271.0 | 4.20 | 0.700 | 41.60 | 160.0 |

^3^ The primary standard level in the Marine Sediment Quality Standard of China (GB 18668-2002);

^4^ The probable effect level (PEL) in the Canadian Sediment Quality Guidelines (CSQG-1999)
